# Global research priorities to accelerate programming to improve early childhood development in the sustainable development era: a CHNRI exercise

**DOI:** 10.7189/jogh.09.020703

**Published:** 2019-12

**Authors:** Mark Tomlinson, Gary L Darmstadt, Aisha K Yousafzai, Bernadette Daelmans, Pia Britto, Sarah L Gordon, Elizabeth Tablante, Tarun Dua

**Affiliations:** 1 Institute for Life Course Health Research, Department of Global Health, Faculty of Medicine and Health Sciences, Stellenbosch University, Cape Town, South Africa; 2 School of Nursing and Midwifery, Queens University, Belfast, UK; 3 Department of Pediatrics, Stanford University School of Medicine, Stanford, California, USA; 4 Department of Global Health and Population, Harvard School of Public Health, Boston, Massachusetts, USA; 5 World Health Organization, Geneva, Switzerland; 6 Early Childhood Development Unit, UNICEF, New York, New York, USA; 7 College of Human Ecology, Cornell University, New York, New York, USA

## Abstract

**Background:**

Approximately 250 million children under the age of five in low and middle-income countries (LMICs) will not achieve their developmental potential due to poverty and stunting alone. Investments in programming to improve early childhood development (ECD) have the potential to disrupt the cycle of poverty and therefore should be prioritised. Support for ECD has increased in recent years. Nevertheless, donors and policies continue to neglect ECD, in part from lack of evidence to guide policy makers and donors about where they should focus policies and programmes. Identification and investment in research is needed to overcome these constraints and in order to achieve high quality implementation of programmes to improve ECD.

**Methods:**

The Child Health and Nutrition Research Initiative (CHNRI) priority setting methodology was applied in order to assess research priorities for improving ECD. A group of 348 global and local experts in ECD-related research were identified and invited to generate research questions. This resulted in 406 research questions which were categorised and refined by study investigators into 54 research questions across six thematic goals which were evaluated using five criteria: answerability, effectiveness, feasibility, impact, and effect on equity. Research options were ranked by their final research priority score multiplied by 100.

**Results:**

The top three research priority options from the LMIC experts came from the third thematic goal of improving the impact of interventions, whereas the top three research priority options from high-income country experts came from different goals: improving the integration of interventions, increasing the understanding of health economics and social protection strategies, and improving the impact of interventions.

**Conclusion:**

The results of this process highlight that priorities for future research should focus on the need for services and support to parents to provide nurturing care, and the training of health workers and non-specialists in implementation of interventions to improve ECD. Three of the six thematic goals of the present priority setting centred on interventions (ie, improving impact, implementation of interventions and improving the integration of interventions). In order to achieve higher coverage through sustainable interventions to improve ECD with equitable reach, interventions should be integrated and not be sector driven.

Child mortality has significantly reduced since 1990. The 62% reduction between 1990 and 2016 has resulted in approximately 5.6 million more children reaching their fifth birthday than would have if 1990 mortality rates had remained the same [1]. Many children who survive continue to live in chronic adversity, extreme poverty, food insecurity and conflict. Approximately 250 million children under the age of five in low- and middle-income countries (LMICs) will not meet their developmental potential due to poverty and stunting alone [2]. There is increasing evidence from both high-income countries (HICs) as well as LMICs that quality interventions that promote healthy growth and development in the early years enhance health equity [3], improve learning and academic attainment [4,5], lower crime and violence levels [4], and in the longer term significantly improve adult health and economic productivity [6]. In contrast, early deficits perpetuate the cycle of poverty and further limit academic performance and opportunities in adulthood even in the next generation [2,7]. Investments in programming to improve early childhood development (ECD) have the potential to disrupt the cycle of poverty and therefore should be prioritised [8].

There has been a marked increase in high-level support for early childhood development (ECD) in recent years [2,9,10]. Three Lancet ECD series [2,7,11], coupled with the Survive, Thrive, and Transform agenda within the Global Strategy for Women’s, Children’s and Adolescents’ Health [12], as well as the inclusion of a specific child development target in the SDGs (goal 4, target 4.2 by 2030 ensure that all girls and boys have access to quality services in the early years,, care, and pre-primary education so that they are ready for primary education), have set the stage for significant progress towards global improvement in ECD outcomes [13]. Finally, the ‘Nurturing Care for Early Childhood Development: A Framework for Helping Children Survive and Thrive to Transform Health and Human Potential’ was launched at the World Health Assembly of the World Health Organization in May 2018 [14]. Despite this multi-lateral support, as well as the substantial evidence base, many countries continue to fall short of SDG target 4.2, with national budgets not making adequate provision for programming in the early years while the policy environment is often not conducive to providing support to improve ECD [15,16]. Shiffman and Smith have shown how for an issue to gain political priority global and local political leaders need to publicly express support, appropriate resources must be allocated, and policies must be enacted to address the issue [16].

One possible reason for the lack of political priority in many countries is a paucity of implementation evidence to guide policy makers and donors about where they should focus their investments in policies and programmes [15]. Identification and investment in research is needed to overcome these constraints. The recent Lancet series “Advancing Early Childhood Development: from Science to Scale” stressed the need for interventions to be implemented as multi-sectoral intervention packages that target multiple risks and that build on existing delivery platforms [17]. The series also highlighted the need for more implementation research to combine interventions into essential packages to ensure feasibility of scale up [17]. In addition, this needs to happen in the context of a supportive environment of policies such as income support, paid leave, breastfeeding breaks at work, childcare and free pre-primary education. These supportive laws provide improved access to quality health services, and importantly money and time that allows caregivers to provide nurturing care for their children [7].

In research to improve ECD, as is the case in many other areas, research needs always exceed available resources [18], and where competition for resources is common, there is a need to set priorities for research investment [19-21]. In most cases, there will never be agreement on which outcomes are preferable and therefore an ethical framework that emphasises the process through which research priorities are set is needed [19,22]. The objective of this paper was to define research priorities for improving ECD to 2025, using the Child Health Research and Nutrition Research Initiative (CHNRI) priority setting methodology [23], as part of a broader initiative to set priorities for maternal, newborn, child, and adolescent health and nutrition within the World Health Organization (WHO).

## METHODS

### Overview of CHNRI approach

The CHNRI research priority setting methodology is a transparent consensus-building tool [23-26] that can be applied at national or global levels and for a variety of purposes, for example to address a single disease, a group of diseases, or risk factors [27]. The CHNRI methodology details a list of individual questions (termed research options) that are independently scored by technical experts against a pre-defined set of criteria. The CHNRI priority setting methodology has been successfully used to set research priorities in a variety of global health domains [18,21,28].

### Establishment of a core group and determination of research criteria

**Figure 1:**
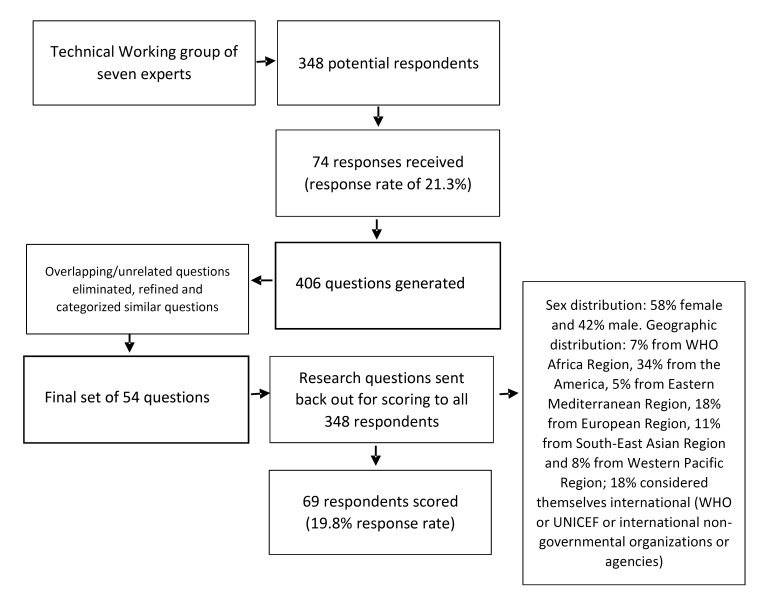
Study flowchart from establishment of a management group to scoring of research priorities.

A group of seven leading global technical experts in the area (TD, MT, ET, PB, AY, BD and GD, primarily working in health sciences) formed a technical working group (TWG). The first step of the research priority setting process involved identifying the context (LMICs using World Bank criteria [29]), time-frame (next 10 years), and target population (children, parents, care providers). The second step involved generating a systematic list of research options. Library searches and snowball sampling were used to identify 348 experts – both researchers and programme experts primarily from the health sciences field – who were approached by email to provide between three to five research options each (Figure 1). Among the group of experts approached, 74 (21.3%) responded with 406 potential research options. The options were then refined (ie, by eliminating redundancies and overlaps and by excluding irrelevant options) and 54 research options were organized by thematic area.

Each of the 54 research options was sent back to the entire group of 348 experts (regardless of whether they participated in the question generation or not) for scoring by answering the questions pertaining to five criteria (**Box 1**) on the following scale: a) no (0 points), b) yes (1 point), or c) not sure (0.5 points). The 69 returned scoring sheets (19.8% response rate) were checked for errors and then scores were entered into a master calculation sheet. In some instances experts may not have felt that they were knowledgeable enough to answer questions about a research option or about particular criteria for a research option. In these cases answers were left blank. The methodology deals with missing answers as it does not expect that each expert has all the necessary knowledge on each possible research option to score it against the criteria [30]. Moreover, according to the *Wisdom of Crowds Theory* [31], it is important to take into account the collective opinion of a group of individuals rather than a single expert (or small number of experts) to answer a question, because it has been shown that the average of collective guesses are nearly always closer to the truth than any single expert judgement. Naturally, these judgements include personal biases but these are cancelled out or at least diluted [26,31]. The pre-requisites for this process to work are:

Box 1 **Criteria the experts applied to each research option.**

**Answerability.** Is it likely that the research question can be answered ethical?

**Effectiveness.** Is it likely that the new knowledge would lead to an effective intervention or program?

**Feasibility.** Is it likely that an answer to this question will lead to/impact health interventions that will be deliverable and feasible within the context?

**Impact.** Is it likely that the intervention or program could improve child health and development substantially?

**Effect on equity.** Is it likely that the intervention or program will reduce inequity? Namely, will it reach and improve the health and development of the most vulnerable groups as well as the more advantaged?

- Diversity of opinion (each person should have private information even if it's just an eccentric interpretation of the known facts);
- Independence (people's opinions are not determined by the opinions of those around them);
- Decentralisation (people are able to specialise and draw on local knowledge); and
- Aggregation (some mechanism exists for turning private judgments into a collective decision – in this case, the CHNRI method).

Intermediate research priority scores were calculated by summing all the answers across the criteria (ie, “1”, “0.5” or “0”). This sum was divided by the number of answers received (blanks were left out of the numerator and the denominator). This resulted in a research priority score (RPS) between 0 and 100% for each research option; the scored research options were then ranked by their final RPS. The RPS represents a score of how much the experts believe that the research option would satisfy the priority setting criteria in Box 1 [30]. RPSs and average expert agreement (AEA) scores were calculated for each research option. Missing (or undecided; “0”) responses meant that a Fleiss Kappa statistic to assess agreement was not appropriate (see [18]). This is in accordance with previous research priority exercises that used the CHNRI methodology [32]. With a large number of scorers and few scoring options, it is not possible to rule out chance when calculating statistical significance for a Fleiss Kappa statistic [32]. The AEA statistic is an average proportion of scorers (mode) that agreed on the five questions asked (**Box 1**). Although the AEA does not give an indication of statistical significance, it was assumed that funders and or policy makers would find it useful as it creates a general overview of the agreement between experts. The AEA was calculated for each research option as follows:


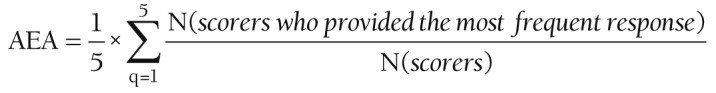


In order to determine differences in scores between respondents from LMIC and those from HIC, responses were stratified by location and a Spearman’s Rho correlation coefficient was calculated to determine the correlation of research option ranks between these groups. Spearman’s Rho correlation determines the degree of correlation between two sets of ranked research options. A correlation coefficient of 1 indicated a high, positive association between two ranked sets; correlation coefficient of -1 indicate a high negative association between two ranked sets, and a correlation coefficient of 0 indicates no association.

## RESULTS

A total of 69 experts participated independently in the voluntary scoring exercise (a 19.8% response rate). Scoring took place over three months. The respondents consisted of 41 (59%) researchers/scientists, 5 programme managers, 3 in the field of public health (self-described), 2 working in policy, 7 working in other fields and 11 (16%) were left blank. Among the respondents, 54% were female, 39% were male (7% non-disclosed), 55% were from HICs and 43% were from LMICs (with one left undisclosed). The final results of the scoring process (top 20 and bottom 10) are shown in **Table 1**.

**Table 1:** Research priority scores and ranks of the 50 research options after application of the CHNRI (Child Health and Nutrition Research Initiative) Methodology to early childhood development in low- and middle-income countries

Importance or potential impact rank (Overall rank)	Research options	Overall RPS	Average expert agreement (AEA)
1	Can child development packages focusing on nurturing care and parent support improve child cognitive development in rural low income settings?	87.34	0.78
2	Can community health workers/paraprofessionals be trained to deliver ECD interventions effectively?	86.29	0.75
3	Can ECD programs be integrated with existing routine health care visits?	86.05	0.75
4	Would the integration of ECD counselling model within an integrated maternal, new-born and child health strategy lead to better child development outcomes?	85.03	0.73
5	What parenting support programs can be developed for low birth weight (LBW) (low for gestational age, premature) and medically at-risk new-borns?	84.83	0.70
6	In what ways can ECD strategies be modified to include and benefit children with disabilities and their families?	84.69	0.71
7	Do combined ECD and income strengthening interventions have an incremental effect on early childhood development?	84.24	0.72
8	Can ECD programmes be taken to scale and maintain the degree of integrity/fidelity necessary to assure effectiveness?	84.02	0.71
9	What are the additive costs of integrating health/nutrition interventions into early childhood education programs?	83.57	0.71
10	What are the most effective models to train parents and members of extended family to provide supportive and effective home learning environments?	82.02	0.68
11	Are group-based interventions more effective than home visiting to deliver ECD interventions?	81.89	0.68
12	What are the most effective behaviour change techniques to optimize parenting skills?	81.64	0.68
13	What approaches to improve quality of early childhood care and education programs result in improved developmental outcomes for young children?	81.29	0.65
14	Can group-based parenting support programs in the postnatal period increase self-efficacy of new mothers?	81.07	0.66
15	What is the impact and sustainability of nutritional supplementation to improve the physical and cognitive health of children?	80.34	0.67
16	What is the comparative cost-effectiveness of home-based care vs centre-based care vs other forms of informal early childhood interventions?	79.85	0.64
17	Can “developmental milestone checks” be included in paediatric visits among children affected by HIV and AIDS in order to improve short and longer term health and developmental milestones?	79.64	0.65
18	What factors contribute to growth and development recovery following early nutritional deficiencies?	79.24	0.67
19	Can a home visiting program delivered by trained community workers prevent child maltreatment?	79.00	0.61
20	What is the impact of father involvement on enhancing the effectiveness of integrated ECD interventions?	78.88	0.62
45	What is the strength of association between stunting and cognitive development?	69.50	0.53
46	Can an e-learning course increase awareness of primary care physicians about early childhood development?	69.24	0.57
47	What are the long term effects of ARVs therapy during pregnancy on child development?	68.88	0.52
48	Do violence prevention programmes that focus on behaviour management improve cognitive stimulation?	68.01	0.49
49	What is the influence of pregnancy planning on parenting behaviours and child development?	67.90	0.50
50	What is the societal and economic impact of developmental disabilities over the life-course?	63.08	0.53
51	Does a behavioural randomized intervention to promote healthier sleep during the first year of life promote neurocognitive development outcomes at ages 12and 24 mo?	58.72	0.50
52	What are best biological indicators to monitor the impact of successful ECD programs?	53.66	0.52
53	What are the genetic and environmental factors that enhance self-monitoring and emotional control?	53.51	0.50
54	What are the gene-environment interactions leading to risk or resilience associated with stressful environments?	52.74	0.46

RPS – research priority score, ECD – early childhood development

The final RPSs for the 54 research options ranged from 52.7 to 87.3, with a median score of 75.2. The average expert agreement scores ranged from 0.46 to 0.78, with a median score of 0.60. Consistent with previous CHNRI exercises, the average expert agreement was significantly and positively correlated (although weak to moderate) with the RPS (*r* = 0.386, *P* < 0.01). Mean scores for each criterion were highly inter-correlated (**Table 2**). Research options were organised by six thematic areas or goals:

**Table 2:** Correlation (Pearson) between mean category scores and total research priority scores across items

	Effectiveness	Feasibility	Impact	Equity	RPS
**Answerability**	0.752*	0.842*	0.696*	0.515*	0.822*
**Effectiveness**		0.923*	0.916*	0.743*	0.957*
**Feasibility**			0.874*	0.684*	0.951*
**Impact**				0.828*	0.956*
**Equity**					0.843*

RPS – research priority score

*P < 0.001.

1. Improve awareness and promotions (#30, 36, 44, 46; numbers indicate the rank position in the main list);
2. Advance identification of risk-factors, and better understanding of the burden (#18, 25, 26, 34, 39, 41, 43, 45, 47, 49, 52, 53, and 54);
3. Improve impact of interventions (#1, 5, 6, 10, 12, 13, 15, 20, 31, 35, 34, 42, 48, and 51);
4. Enhance implementation of interventions (#2, 11, 14, 19, 24, 27, 28, 32, and 38);
5. Expand integration and coordination (#3, 4, 8, 17, and 23); and
6. Increase understanding of health economics and social protection strategies (#7, 9, 16, 21, 22, 29, 33, 40, and 50).

The top three ranked research options were (**Table 3**):

**Table 3:** The top 3 research options (and their ranking) for each thematic area or goal

	Ranking
**Improve awareness and promotion:**	
What is the impact of demand side strategies designed to reduce access barriers for poor and vulnerable groups on pre-primary enrolment?	30
What are cost-effective ways to promote an understanding of child development at the community?	36
What is the impact of social mobilization campaigns on use of positive discipline?	44
**Advance identification of risk-factors, and better understanding of the burden:**	
What factors contribute to growth and development recovery following early nutritional deficiencies?	18
What tools can be used at the community level for early identification of developmental disorders?	25
What are the most appropriate tools for population level assessment of development in children -8 y in resource limited settings at scale?	26
**Improve impact of interventions:**	
Can child development packages focusing on nurturing care and parent support improve child cognitive development in rural low income settings?	1
What parenting support programs can be developed for LBW (low for gestational age, premature) and medically at-risk newborns?	5
In what ways can ECD strategies be modified to include and benefit children with disabilities and their families?	6
**Enhance implementation of interventions:**	
Can community health workers/paraprofessionals be trained to deliver ECD interventions effectively?	2
Are group based interventions more effective than home visiting to deliver ECD interventions?	11
Can group-based parenting support programs in the postnatal period increase self-efficacy of new mothers?	14
**Expand integration and coordination:**	
Can ECD programs be integrated with existing routine health care visits?	3
Would the integration of ECD counselling model within an integrated maternal, new-born and child health strategy lead to better child development outcomes?	4
Can ECD programmes be taken to scale and maintain the degree of integrity/fidelity necessary to assure effectiveness?	8
**Increase understanding of health economics and social protection strategies:**	
Do combined ECD and income strengthening interventions have an incremental effect on early childhood development?	7
What are the additive costs of integrating health/nutrition interventions into early childhood education programs?	9
What is the comparative cost-effectiveness of home-based vs centre-based vs other non-formal early childhood interventions?	16

ECD – early childhood development

1. “Can child development packages focusing on nurturing care and parent support improve child cognitive development in rural low income settings?” (Improve impact of interventions)
2. “Can community health workers/paraprofessionals be trained to deliver ECD interventions effectively?” (Enhance implementation of interventions)
3. “Can ECD programs be integrated with existing routine health care visits?” (Expand integration and coordination)

### Scores by region, sex and occupation

In order to explore differences between groups, a number of other comparisons were made based on whether experts were from a LMIC or HIC, sex and their occupation. Scores from respondents in LMICs (mean = 75.6; range = 54.5-88.4) were much higher than their HIC expert group counterparts (mean = 61.2; range = 43.1-78.3) (**Table 4** and **Table 5**). The Spearman’s Rho correlation of the research options ranks between LMIC experts and HIC experts was close to zero, and statistically non-significant (Spearman’s r = -0.001, *P* > 0.05).

**Table 4:** The top 5 research priority scores and ranks from experts in LMIC

LMIC rank	Research option	LMIC RPS	LMIC AEA	Overall rank
**1**	What parenting support programs can be developed for LBW (low for gestational age, premature) and medically at-risk new-borns?	88.4	61.6	5
**2**	Can child development packages focusing on nurturing care and parent support improve child cognitive development in rural low income settings?	87.6	64.4	1
**3**	What are the most effective behaviour change techniques to optimize parenting skills?	86.3	61.7	12
**4**	Can community health workers/paraprofessionals be trained to deliver ECD interventions effectively?	86.1	60.8	2
**5**	Would the integration of ECD counselling model within an integrated maternal, new-born and child health strategy lead to better child development outcomes?	86.1	60.4	4

LMIC – low- and middle-income country, AEA – average expert agreement, LBW – low-birth weight, ECD – early childhood development

**Table 5:** The top 5 research priority scores and ranks from experts in HIC

HIC rank	Research option	HIC RPS	HIC AEA	Overall Rank
**1**	Can ECD programs be integrated with existing routine health care visits?	90.4	78.3	3
**2**	What are the additive costs of integrating health/nutrition interventions into early childhood education programs?	87.2	77.8	9
**3**	Can child development packages focusing on nurturing care and parent support improve child cognitive development in rural low income settings?	87.1	77.3	1
**4**	In what ways can ECD strategies be modified to include and benefit children with disabilities and their families?	86.5	75.7	6
**5**	Can community health workers/paraprofessionals be trained to deliver ECD interventions effectively?	86.4	74.9	2

HIC – high income country, RPS – research priority score, AEA – average expert agreement, ECD – early childhood development

Responses from experts were also stratified by sex in order to determine any differences between groups (**Table 6** and **Table 7**). The female expert group scores (mean = 76.5; range = 52.2-88.3) were slightly higher than the male expert group counterparts (mean = 72.7; range = 45.2-87.2). The Spearman’s Rho correlation of the research options ranks between female experts and male experts was positive, weak and statistically significant (Spearman’s r = 0.272, *P* < 0.05).

**Table 6:** The top 5 research priority scores and ranks from female experts

Female rank	Research option	Female RPS	Female AEA	Overall rank
**1**	Can ECD programs be integrated with existing routine health care visits?	88.3	80	3
**2**	Can ECD programmes be taken to scale and maintain the degree of integrity/fidelity necessary to assure effectiveness?	88	77.2	8
**3**	Can child development packages focusing on nurturing care and parent support improve child cognitive development in rural low income settings?	87.7	78.9	1
**4**	Do combined ECD and income strengthening interventions have an incremental effect on early childhood development?	87.1	76.2	7
**5**	In what ways can ECD strategies be modified to include and benefit children with disabilities and their families?	85.9	73.3	6

RPS – research priority score, AEA – average expert agreement, ECD – early childhood development

**Table 7:** The top 5 research priority scores and ranks from male experts

Male rank	Research option	Male RPS	Male AEA	Overall rank
**1**	Can child development packages focusing on nurturing care and parent support improve child cognitive development in rural low income settings?	87.2	77.9	1
**2**	Can community health workers/paraprofessionals be trained to deliver ECD interventions effectively?	87.1	74.8	2
**3**	Would the integration of ECD counselling model within an integrated maternal, new-born and child health strategy lead to better child development outcomes?	86.2	73.7	4
**4**	What parenting support programs can be developed for LBW (low for gestational age, premature) and medically at-risk new-borns?	85.4	70.8	5
**5**	What are the additive costs of integrating health/nutrition interventions into early childhood education programs?	82.8	68.4	9

RPS – research priority score, AEA – average expert agreement, ECD – early childhood development

Furthermore, responses from experts were stratified by occupations (ie, academics vs non-academics; non-academics included those working in programme management, policy, as a health practitioner, other (including blanks) in order to determine any differences between groups of occupations (**Table 8** and **Table 9**). The academic expert group’s (59%) scores (mean = 75.3; range = 51.2-87.1) were similar to their non-academic expert group (41%) counterparts (mean = 75.1; range = 46.9-89.2). The Spearman’s Rho correlation of the research questions ranks between academic experts and non-academic experts was close to zero, and statistically non-significant (Spearman’s r = -0.001, *P* > 0.05).

**Table 8:** The top 5 research priority scores and ranks from academic experts

LMIC rank	Research question	Academic RPS	Academic AEA	Overall rank
**1**	Can ECD programs be integrated with existing routine health care visits?	87.1	0.76	3
**2**	Can community health workers/paraprofessionals be trained to deliver ECD interventions effectively?	86.9	0.74	2
**3**	Can child development packages focusing on nurturing care and parent support improve child cognitive development in rural low income settings?	86.7	0.76	1
**4**	Would the integration of ECD counselling model within an integrated maternal, new born and child health strategy lead to better child development outcomes?	85.8	0.74	4
**5**	Do combined ECD and income strengthening interventions have an incremental effect on early childhood development?	84.1	0.7	7

LMIC – low- and middle-income country, RPS – research priority score, AEA – average expert agreement, ECD – early childhood development

**Table 9:** The top 5 research priority scores and ranks from non-academic experts

HIC rank	Research question	Non-academic RPS	Non-academic AEA	Overall rank
**1**	What parenting support programs can be developed for LBW (low for gestational age, premature) and medically at-risk new-borns?	89.2	0.8	5
**2**	Can child development packages focusing on nurturing care and parent support improve child cognitive development in rural low income settings?	88.2	0.79	1
**3**	In what ways can ECD strategies be modified to include and benefit children with disabilities and their families?	87.7	0.77	6
**4**	What are the additive costs of integrating health/nutrition interventions into early childhood education programs?	85.6	0.73	9
**5**	Can community health workers/paraprofessionals be trained to deliver ECD interventions effectively?	85.4	0.76	2

HIC – high income country, RPS – Research Priority Score, AEA – average expert agreement, LBW – low-birth weight, ECD - early childhood development

## DISCUSSION

A diverse range of global health sciences experts with knowledge and experience in the field engaged in the well-established CHNRI methodology to identify research priorities for improving ECD to 2025. The systematic ranking of research options against predetermined criteria exposes the strengths and weaknesses of the research options and offers greater replicability and transparency than other priority setting methods [33]. The results of this process highlight that experts’ priorities for future research focus on testing the best models of service and support to parents to enable them to provide nurturing care, as well as research on how to train health workers and non-specialists in the effective implementation of interventions to improve ECD. Sixty-nine experts eventually undertook scoring for this priority setting exercise. The more experts who agree to participate in the scoring, the more reliable the outcomes of the research priority setting exercise [30]. The range of scores (both RPS and AEA) were consistent with previous CHNRI exercises. The RPS range shows significant variation, indicating that the methodology discriminated well among the competing research options.

Three of the six thematic goals of the present priority setting centered on interventions (ie, improving impact, implementation of interventions and improving the integration of interventions). These three goals have the highest average RPSs. In order to achieve higher coverage through sustainable high quality interventions to improve ECD i with equitable reach, interventions should be integrated into health, nutrition, education and child protection sectors [7,34]. This is in line with the Nurturing Care Framework (NCF) guiding principles of a whole of-society approach including all sectors [14]. However, there is limited knowledge on how to best integrate programmes to improve ECD [35,36]. The NCF offers an important framework to help move this forward. In addition, the Lancet Commission on Child Health and Well-being aims to place the child at the centre of all country actions and will stress how attempts to cultivate healthy productive societies must begin with investment in children [37].

Another key finding of this exercise was that simply increasing access to programmes in the early years is not sufficient unless programme quality is also improved [38,39]. The theme with the highest rank (improving the impact of interventions) is specifically aimed at quality assurance though testing and implementing best models. This is in line with the current global focus on the importance of quality at the level of programmes as well as at the level of the health system [40,41].

The second highest scoring option “Can community health workers/paraprofessionals be trained to deliver ECD interventions effectively?” addresses human resource skill and capacity, and is a reflection of the global shortage (but especially in LMICs) of skilled health workers to implement interventions to improve ECD. This finding is consistent with the barriers to scale up in ECD research identified by Richter et al. [7] and also with the priority setting exercise of Sharma and colleagues [8], which lends further credibility to the results of the present priority setting exercise. Seven of the 54 research priorities focused on the financial aspects of programmes and interventions (most of these came from the sixth goal: increasing the understanding of health economics and social protection strategies) although these research priority options were not highly ranked [7], and given the range of experts it was somewhat surprising that these research options were not ranked higher. It should be noted however, that the difference in score between the highest scoring research option and the first finance-related question (score of 79.85 and a rank of 16) was only 7.5 percentage points. The aim of the CHNRI exercise is to produce a transparent account of the scoring (the collective thinking) of a range of experts, that then permits funding agencies to make decisions with some level of data and ability to understand where one option stands in comparison to others. An agency might then decide to fund options 5 and 16, for example, based on their priorities and having seen a small difference between the top 5 ranked options and those in places 10-16.

In order to compare responses by sub-groups, comparative analyses were undertaken by geographic location, sex and by occupation (ie, academic vs non-academic). There was almost no agreement between experts from LMIC (44%) and experts from HIC (56%). This is perhaps unsurprising given the vast variation in types of programmes to improve ECD, sectors, implementers and target populations globally [38]. Experts in the LMIC group gave significantly higher scores overall than experts from HIC. The top three research priority options from the LMIC experts came from the third thematic goal of improving the impact of interventions, whereas the top three research priority options from the HIC experts came from different goals: improving the integration of interventions and increasing the understanding of health economics and social protection strategies, as well as improving the impact of interventions. Experts from LMICs were in agreement with HIC respondents about the need to prioritise impact with high quality interventions [38].

Female (58%) experts gave slightly higher scores than their male (42%) counterparts. There was moderate agreement between female and male experts. The top three research priority options from the female and male experts were from the goals of improving ECD interventions. The academic expert group (ie, working in research; 59%) gave a slightly larger range of scores compared to the non-academic expert group (ie, programme management, policy, health practitioner, other or those left blank; 41%). In addition, there was little specific agreement between academic experts and non-academic experts. The highest rated research priority from the academic expert group came from the fifth thematic goal of expanding the integration and coordination of programmes targeting the early childhood period, and the second highest came from the fourth thematic goal of enhancing the implementation of interventions. The top three research priorities from the non-academic group of experts came from the third research priority thematic goal of improving the impact of interventions. In general, however, the top three thematic goals as rated by the academics and non-academics came from a set of goals that centred on improving interventions (ie, either improving impact, implementation or integration). Ensuring high quality implementation of interventions is a particular concern currently in global health [38,39].

In this priority setting exercise the questions that were proposed varied from the general (the highest ranked option: child development packages focusing on nurturing care to improve child cognitive development) to the specific (how sleep promotion can improve neurocognitive development, Rank 51). This may in part be due to the nature of the respondents, but could also be a function of the nature of the methodology – in particular the two criteria of ‘impact’ and ‘effect on equity.’ General questions, with the promise of greater impact and for reducing inequity are likely to be ranked higher.

The CHNRI methodology has a number of advantages over other priority setting approaches [30]. The systematic listing and scoring of research options limits the influence of personal bias, while there is transparency in the input and contribution of individuals involved in scoring research priorities. The method also prevents one or a small group of individuals from dominating the process, while also providing a quantitative measure. The CHNRI methodology also has the added advantage of including non-technical stakeholders, and different domains of research can be simultaneously evaluated using the same set of criteria [30]. Strengths of our priority setting exercise include using a proven methodology, a sufficient number of experts with an acceptable gender balance, geographical spread and occupational balance.

Limitations to the approach undertaken in this study relate to the validity (it is not yet possible to verify the validity of the rankings) [42] of the CHNRI approach overall, and the potential sampling biases and high non-response rate [32,42]. While 74 people participated in generating the research options that were ultimately rated, it is unattainable in such an exercise to create a set of research options that comprehensively covers all possible research options. As a result, both the research options and the ratings generated are likely to replicate biases of the participating experts. In addition, participants not fluent in English would have limited participation in such an exercise. It is conceivable that the scores of experts who responded could be systematically different from those who did not respond [32]. In addition, the experts who did respond were primarily from the health sciences. The utility of future priority setting exercises may be improved through research to explore how the variability of profession among the raters in a CHNRI exercise affects both the research options provided as well as the final scoring. Different strategies to recruit a more diverse panel of experts need to be considered and put in place in future exercises.

The low response rate likely was a result of the time consuming nature of the exercise, but our response rate, while lower, was not markedly different from other similar exercises [21,26,43]. Dean and colleagues [43] conducted a priority setting exercise on preconception care in LMICs to reduce maternal and child mortality and morbidity with a 37% response rate, while George et al. [44] had a response rate of 36% in a priority setting exercise on reducing preterm births and stillbirths. However, the CHNRI methodology attempts to account for this based on the theory of the wisdom of crowds that suggests that approximately 24 scorers are needed in order to cancel out personal biases and judgements and to arrive at the collective wisdom of the group [31].

Finally, in the light of current global priorities, a number of key areas of research are notable for their absence in this exercise. Despite early childhood care being a key global issue there was only one research option on childcare (Rank 13: approaches to improve early childhood care). Equally surprising is that there were no questions (to be scored) on the delivery or implementation of interventions in humanitarian settings or fragile states. Given the current global crises in terms of children in humanitarian settings and future concerns in this regard, this is a significant limitation of this exercise. Also, current initiatives to develop population-based, cross-country measurement tools of child development did not feature in the research priorities of this group.

Despite the above limitations, this study was successful in producing research options from 74 experts, and scores from 69 global experts. In addition, this study demonstrates a need to invest in research that focuses on improving the impact, implementation and integration of interventions to improve ECD.

## CONCLUSION

This research priority setting exercise provides a significant contribution to establishing research priorities in the field of ECD to 2025. International organisations, national governments, research institutes, and donors are encouraged to consider the findings of this exercise in order to address key gaps in our knowledge and enhance the nurturing care for early childhood development agenda and the achievement of the SDGs. Future exercises should ensure a more diverse panel of experts.
